# Overcoming off-target optical stimulation-evoked cortical activity in the mouse brain *in vivo*

**DOI:** 10.1016/j.isci.2024.111152

**Published:** 2024-10-15

**Authors:** Simon Weiler, Mateo Velez-Fort, Troy W. Margrie

**Affiliations:** 1 Sainsbury Wellcome Centre for Neuronal Circuits and Behavior, University College London, 25 Howland Street, London W1T 4JG, UK

**Keywords:** Natural sciences, Biological sciences, Neuroscience, Systems neuroscience, Techniques in neuroscience

## Abstract

Exogenous opsins allow for *in vivo* interrogation of brain circuits at unprecedented temporal and spatial precision. Here, we found that optical fiber laser stimulation at wavelengths of 637, 594, or 473 nm within the cortex of mice lacking expression of exogenous opsins resulted in a strong neuronal response in the contralateral visual cortex. Evoked responses were observed even at low laser intensities (fiber tip power 1 mW) and most pronounced at 637 nm. We took advantage of retinal light adaptation by using a dim external light source (20 lux) that abolished the 594 and 473 nm-evoked neuronal responses even at high laser intensities (15 mW). The prevention of 637 nm-evoked responses, however, could only be achieved for stimulation intensities ≤ 2.5 mW. This highlights the need for careful selection of light wavelengths and intensities for optogenetic experiments. Additionally, retinal light adaptation offers an effective solution to minimize unintended activation.

## Highlights

• Optical stimulation within the mouse brain produces off-target cortical activation
• The degree of off-target activation is wavelength and intensity dependent
• Red wavelengths have the strongest off-target effect
• Retinal adaptation via ambient light overcomes laser-evoked off target responses

## Graphical abstract

**Figure:**
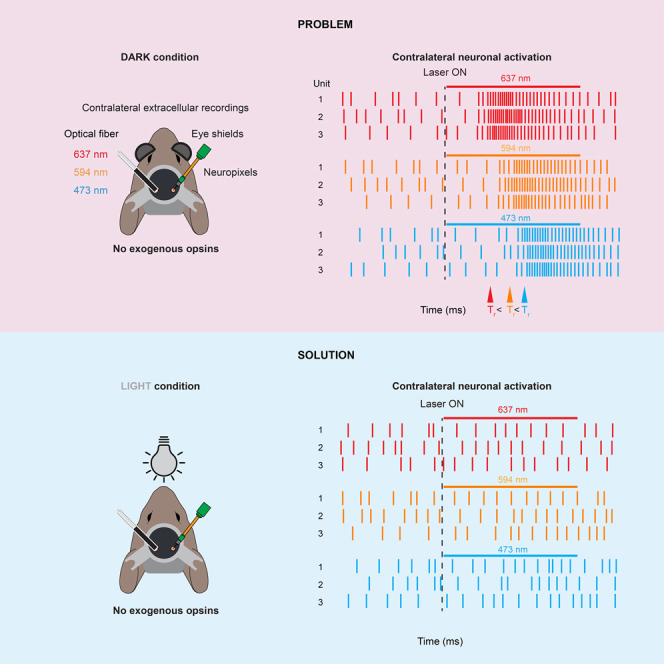

## Introduction

Optogenetic tools have revolutionized the field of *in vivo* systems neuroscience by offering temporally precise, reversible, and cell type-specific modulation of neural activity in the brain.^1^^,^^2^^,^^3^^,^^4^^,^^5^^,^^6^ For example the selective activation or silencing of populations of cells expressing genetically encoded wavelength-specific opsins has revealed important causal roles of neuronal circuits underlying animal behaviors.^7^^,^^8^ More recently, short (473 nm) and longer wavelength-sensitive (594 nm and 637 nm) opsins have been used to independently modulate the activity of non-overlapping neuronal populations within the same brain area^9^^,^^10^^,^^11^ and expression of dual-color opsins even enable both activation and suppression of activity within the same neurons.^12^ In combination with the use of optical fiber implants these tools allow for spatially restricted stimulation of neurons in deep brain areas^4^^,^^13^^,^^14^ and thus the dissection of the role of excitation and inhibition within specific circuits during behavior.

Although the delivery of light from within the brain allows the interrogation of circuits in deep structures, it is crucial to consider the non-specific effects of light propagation through brain tissue when performing optogenetic experiments—in particular, when using high light intensities and red-shifted wavelengths. For example, electroretinogram (ERG) recordings indicate that the retina can be directly activated by red, orange, and blue light emitted from the tip of the optical fiber implanted within the brain at laser light intensities found to disrupt behavioral tasks in mice.^15^ From a behavioral standpoint such off-target retinal activation-induced artifacts may be overcome using light adaptation that decreases the sensitivity of the retina to wavelengths of light used for optogenetic stimulation.^15^ Importantly, and despite rodents being considered dichromats (highest sensitivity at ultraviolet and green light), off-target activation of the retina has been found to be most prominent when using red light.^15^ This is explained not only by recent evidence showing that rodents are not red-light blind^16^ but also by the increased penetration of long-wavelength light through brain tissue when compared to short wavelengths.^15^^,^^17^^,^^18^^,^^19^^,^^20^

While the risk of off-target activation of retinal opsins has been shown to lead to behavioral artifacts,^15^ its impact on distant downstream neuronal circuits remains unknown. Moreover, the extent to which retinal light-adaptation using external ambient illumination impacts downstream brain activity is largely unexplored. In this study, we tested the wavelength- and intensity-dependent effect of retinal activation by optical stimulation within the brain on cortical neuronal responses using Neuropixels recordings in the absence of expression of any exogenous opsins. We find that while red, orange, and blue light emitted deep within the cortex can cause pronounced modulation of contralateral visual cortical activity this may be ameliorated by the presence of an external ambient light source.

## Results

### In complete darkness and in the absence of exogenous opsins optical-fiber illumination within the brain activates the mouse visual cortex

**Figure 1:**
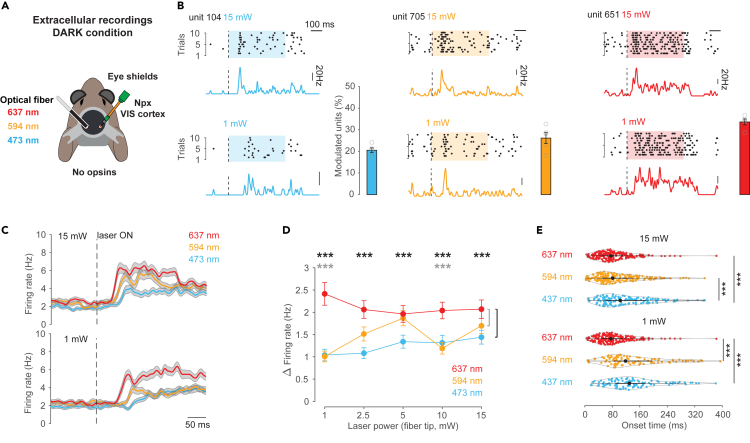
Optical-fiber illumination in the brain using different wavelengths strongly activates the mouse visual cortex in darkness (A) Schematic of the recording setup. Red (673 nm), orange (594 nm), or blue (473 nm) laser was delivered via an optical fiber implanted in the visual cortex (VIS, left hemisphere) of awake head-fixed mice. Translaminar Neuropixels recordings were performed in the contralateral VIS in complete darkness. Black dental cement in and around the headplate and blackout adhesive around the optical fiber were used to prevent light emitting through the craniotomy, skull and optical fiber. In addition, eye cups were gently positioned to touch the fur around the eye to further ensure there to be no external activation of the retina. (B) Raster plots of spikes of three individual units in response to ten repetitions of 0.5 s of blue (left), orange (middle), or red (right) laser stimulation and the corresponding average peri-stimulus time histogram (PSTH). Recordings were performed in complete darkness with laser power of 15 or 1 mW. Bar plots display the average percentage of the total number of units (*n* = 761) activated by blue, orange and red laser stimulation across 1, 2.5, 5, 10, and 15 mW. Open circles indicate the percentage of responding units for each laser intensity. Error bars represent the sem of the percentage of responders over the five laser intensities. (C) Average spike density functions for all units aligned to the onset (dotted line) of red, orange or blue laser stimulation (*n* = 761 cells, *n* = 4 mice) using either 15 mW (top) or 1 mW (bottom). Gray shaded area indicates sem. (D) Average difference in firing rates (mean ± sem) between a baseline window (0.2 s before stimulation) and a response window (0.5 s after onset of stimulation) for red, orange, or blue laser stimulation at different laser power. Asterisks indicate significant differences (p < 0.001) between red and blue (black) as well as red and orange (gray). (E) Violin plots displaying onset latencies of red, orange and blue laser-evoked responses at 15 mW (top) and 1 mW (bottom) laser stimulation. Individual data points (circles), median (black circle) and first and third quartiles are displayed (black lines). Asterisks indicate significant differences (p < 0.001).

We first tested whether laser stimulation of the visual cortex at power intensities/irradiances (1/31.8; 2.5/79.6; 5/159.1; 10/318.2; 15/477.3 mW/mW/mm^2^) and wavelengths (637, 594, and 473 nm) regularly used for *in vivo* optogenetic experiments^6^^,^^15^^,^^21^^,^^22^^,^^23^^,^^24^^,^^25^ had an effect on neuronal activity in the absence of genetically expressed exogenous opsins.^15^ For this, we implanted a small-diameter optic fiber in deep layers of the visual cortex (*n* = 4 mice) and performed Neuropixels recordings in the contralateral visual cortex of head-fixed awake mice under conditions of total darkness (distance between Neuropixels and optic fiber: ∼4 mm, Figure 1A). Importantly, to avoid direct external stimulation of the retina via light emission from the fiber, the ferrule sleeve of the optical fiber was shielded with blackout material. To prevent light being emitted through the brain and skull, black dental cement was used to seal the craniotomy within and around the 1 × 1.2 cm headplate.^26^ Finally, two eye cups were also positioned over the eyes (Figure 1A).

Under these conditions we found many individual units that changed their firing rate in response to laser stimulation using 473, 594, and 637 nm at both high (e.g., 15 mW) and low (e.g., 1 mW) laser intensities (Figures 1B and S1, significance assessed by ZETA test, see STAR Methods). Overall, 18–37% of units responded to laser stimulation across all wavelengths and laser intensities tested (Figure 1B). When averaging across all cells and animals, all wavelengths and stimulus intensities were found to evoke a significant increase in the firing rate of the contralateral visual cortex (e.g., **15 mW**: 473 nm baseline = 2.93 ± 0.2 Hz versus laser ON = 5.16 ± 0.33 Hz; 594 nm baseline = 3.41 ± 0.23 Hz versus laser ON = 5.99 ± 0.37 Hz; 637 nm baseline = 3.29 ± 0.2 Hz versus laser ON = 5.99 ± 0.56 Hz; n = 761 units, *p* < 0.001, **1 mW**: 473 nm baseline = 3.28 ± 0.24 Hz versus laser ON = 5.09 ± 0.33 Hz; 594 nm baseline = 3.51 ± 0.23 Hz versus laser ON = 5.04 ± 0.31 Hz; 637 nm baseline = 3.24 ± 0.21 Hz versus laser ON = 6.6 ± 0.44 Hz; n = 761 units, *p* < 0.001, permutation test, respectively, Figures 1C and 1D).

### Red wavelength light causes the strongest modulation of neuronal activity

By pooling across laser intensities we found that red wavelength light reliably evoked responses in the largest fraction of units (range of percentage of responsive units: red, 29–37%; orange, 18–33%; blue, 18–24%; *p* < 0.05, one-way ANOVA with Tukey-Kramer multiple comparison corrections, Figures 1B and S2). Not only was red wavelength stimulation found to recruit the largest fraction of cells but also most consistently evoked the largest change in firing rate (Figure 1D). Already at the lowest laser intensity, 637 nm light illumination led to the strongest change in firing rates when compared to 594 and 473 nm (Δ firing rate 637 nm = 2.41 ± 0.26 versus Δ firing rate 594 nm = 1.01 ± 0.12 and Δ firing rate 473 nm = 1.04 ± 0.13, *p* < 0.001, Friedman’s test with Tukey-Kramer multiple comparison corrections, Figure 1D).

Consistent with the known scattering properties of these three light wavelengths, we also observed that the onset latencies of the neuronal responses to 637 and 594 nm laser stimulation at 15 mW were significantly shorter compared to 473 nm (latency 637 nm = 87.5 ± 4.038 ms and 594 nm = 98.72 ± 4.94 ms versus latency 473 nm = 121.96 ± 6.36 ms, *n* = 265, 249, 184 units, respectively; *p* < 0.01, Kruskal-Wallis test with Tukey-Kramer multiple comparison corrections; Figure 1E). At 1 mW laser stimulation intensity, 637 had the shortest latency compared to both 594 and 473 nm (latency 637 nm = 111.4 ± 4.69 ms versus latency 594 nm = 144.3 ± 8.9 ms and latency 473 nm = 141.93 ± 7.66 ms, *n* = 199, 100, 102 units, respectively, *p* < 0.01, Kruskal-Wallis test with Tukey-Kramer multiple comparison corrections, Figure 1E).

### External ambient illumination reduces off-target light-evoked neuronal activation

**Figure 2:**
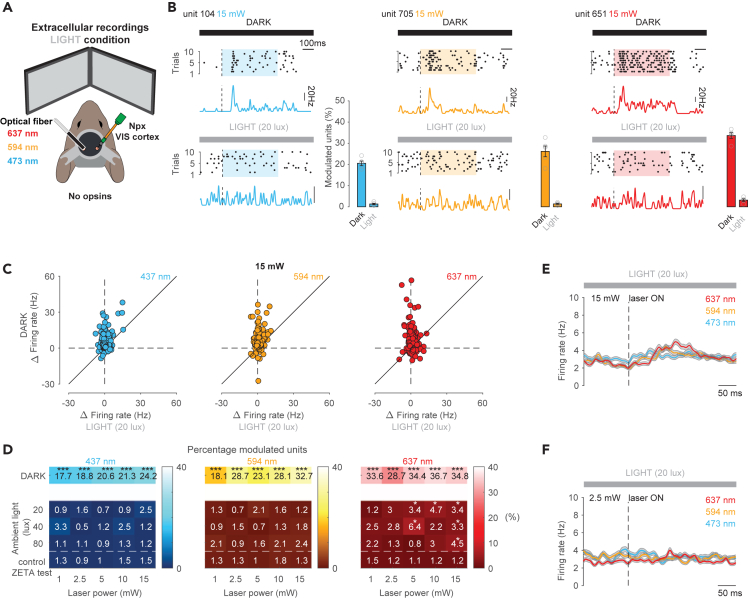
External ambient illumination eliminates internal light-evoked neuronal activation in the visual cortex (A) Schematic of the recording setup. Red (673 nm), orange (594 nm), or blue (473 nm) laser was delivered via an optical fiber implanted in the visual cortex (VIS, left hemisphere) of awake head-fixed mice. Translaminar Neuropixels recordings were performed on the contralateral VIS. External illumination was delivered via two monitors displaying a full screen iso-illuminant gray image. (B) Raster plots of spikes of three individual units (same cells as in Figure 1B) in response to ten repetitions of 0.5 s of blue (left), orange (middle), or red (right) laser stimulation and the corresponding average peri-stimulus time histogram (PSTH). Recordings were performed either in complete darkness with laser power of 15 or 1 mW or under ambient light conditions (indicated by the gray bar). Bar plots display the average percentage of the total number of units (*n* = 761 units, *n* = 4 mice) activated by blue, orange, and red laser stimulation across 1, 2.5, 5, 10, and 15 mW under darkness or ambient light conditions. Open circles indicate the percentage of responding units for each laser intensity. Error bars represent the sem of the percentage of responders over the five laser intensities. (C) Scatterplot displaying the average laser-evoked firing rate change for those cells that responded under darkness versus ambient light conditions for blue (*n* = 184), orange (*n* = 249), red (*n* = 265) laser stimulations at 15 mW. (D) Heatmaps displaying the percentage of units modulated by blue (left), orange (middle) and red (right) laser stimulation under dark (top row) and different ambient light conditions and laser powers. Bottom row displays the false positive percentage for pooled dark and ambient light conditions according to the ZETA test used to assess significant modulation during a control period when laser stimulation was absent. Asterisks indicate significant differences (∗∗∗ p < 0.001, ∗ p < 0.05) between percentages of modulated units under stimulation periods (rows 1–4) compared to baseline periods without any laser stimulation (row 5). (E) Average spike density functions for all units aligned to the onset (dotted line) of red, orange, or blue laser stimulation (*n* = 761 cells, *n* = 4 mice) using 20 lux of ambient light brightness and 15 mW laser stimulation intensity. Gray shaded area indicates sem. (F) Same as E for 2.5 mW laser stimulation intensity.

To determine whether decreasing the sensitivity of the retina could diminish these observed laser-evoked responses in visual cortex we next positioned two monitors in front of the animal and displayed full-field gray iso-illumination at different brightness levels (20, 40, or 80 lux) during laser stimulation (Figure 2A).

Under these conditions we found that very few units responded to blue, orange, and red laser stimulation at laser intensities between 1 and 15 mW in the presence of 20 lux external illumination (Figures 2B–2D and S2). At 15 mW, even many of the units that were activated by red laser stimulation in darkness significantly reduced their laser-evoked firing in the presence of ambient light (Figures 2B, 2C, and S2A, 637 nm Δ firing rate dark = 5.36 ± 0.51 versus Δ firing rate 20 lux = 0.92 ± 0.16, n = 265 units, *p* < 0.001, two-sided Wilcoxon signed-rank test). At a population level, when comparing baseline firing rates to that recorded during laser stimulation, we found no significant increase in neuronal activity at 15 mW when using 594 and 473 nm wavelengths in the presence of ambient light (20 lux; 594 nm firing rate baseline = 3.4 ± 0.17 Hz versus firing rate laser ON = 3.9 ± 0.17 Hz; 473 nm firing rate baseline = 3.85 ± 0.19 Hz versus firing rate laser ON = 4.16 ± 0.21 Hz, *n* = 761; *p* > 0.05, permutation test, respectively, Figure 2E). We also found that the proportion of cells that were activated by laser stimulation at 15 mW greatly decreased (594 nm ambient darkness: 32.7% or 249/761 versus ambient light: 1.2% or 9/761; 473 nm ambient darkness: 24.2% or 184/761 versus ambient light: 2.5% or 19/761; *p* < 0.001, Fisher exact test, Figure 2D). Similar observations were made across all laser intensities and ambient light brightness conditions (Figure 2D). Generally, the proportion of remaining cells still significantly modulated by 473 and 594 nm laser stimulation under the different laser and ambient light conditions were indistinguishable to the false positive rate detected with the ZETA test during a control period without any laser stimulation (Figure 2D).

While the majority of cells that were activated upon 637 nm laser stimulation at 15 mW in complete darkness no longer responded in the presence of ambient light (673 nm ambient darkness: 34.8% or 265/761 versus ambient light: 3.4% or 26/761; *p* < 0.001, Fisher exact test; Figure 2D), we found that some cells maintained their responsiveness. Indeed, 3.4% of units recorded under ambient light conditions and laser stimulation (at 15 mW) was still significantly higher than the false positive proportion detected with the ZETA test during a control period where laser stimulation was absent (673 nm ambient light: 3.4% or 26/761 versus control: 1.2% or 9/761; *p* < 0.001, Fisher exact test, Figure 2D). This small fraction of cells responded significantly enough to cause an average population increase in activity at 15 mW (637 nm firing rate baseline = 3.51 ± 0.2 Hz versus firing rate laser ON = 4.22 ± 0.2 Hz, n = 761 units, *p* < 0.05, permutation test, Figure 2E). Only at laser powers equal or less than 2.5 mW at 20–80 lux ambient light brightness, was the proportion of laser-activated units found to be indistinguishable to the false positive rate (673 nm ambient light: 3% or 23/761 versus control: 1.2% or 9/761; *p* < 0.001, Fisher exact test, Figure 2D) and the average population did not significantly increase in activity upon laser stimulation (637 nm firing rate baseline = 3.5 ± 0.2 Hz versus firing rate laser ON = 3.92 ± 0.2 Hz, n = 761 units, *p* > 0.05, permutation test; Figures 2F and S2A).

Taken together, these results show that in darkness, optical-fiber stimulation within the mouse brain internally activates the retina and subsequently the visual cortex and that while ambient light can prevent off-target retinal activation over a range of experimentally relevant laser intensities for orange and blue light, it does not completely abolish responses to red light at high laser power.

## Discussion

In this study, we found that in darkness, delivery of red, orange, and blue light through optical fibers in live brain tissue lacking exogenous opsins is sufficient to robustly activate cortical neurons. Surprisingly, we find that this unintended neuronal activation is triggered even at the lowest levels of laser intensities commonly used in *in vivo* optogenetic experiments.^6^^,^^15^^,^^21^^,^^22^^,^^23^^,^^24^^,^^25^

In order to exclude the possibility this might be due to potential external activation of the eye via light leakage from the craniotomy itself, we shielded the eyes with eye cups in darkness, covered the dorsal surface of the skull, and craniotomy with black dental cement and used a blackout sleeve around the fiber connector. The simplest explanation for the light-evoked increase in neuronal activity observed in the absence of exogenous opsins is due to off-target activation of endogenous opsins within the retina. In this scenario, light from the fiber tip propagates through the brain tissue,^15^^,^^17^^,^^18^^,^^19^^,^^20^ reaching the back of the eye at intensities that are sufficient to activate retinal photoreceptors and therefore subsequent visual pathways. This idea is supported by periorbital ERG recordings showing that light from implanted optical fibers in the somatosensory cortex can be detected by the retina.^15^ Given the interhemispheric cortical distances between the optical probe and the recording electrodes and the long latencies to response onset, our data cannot be explained by any photoelectric^27^ or intracranial heating^28^^,^^29^^,^^30^ effect that display onset times in the millisecond range. Furthermore, it is difficult to reconcile how external illumination could overcome these previously described non-specific responses^28^^,^^29^^,^^30^ to intracranial laser stimulation.

While it is the case that all three wavelengths tested here led to the unintended activation of neurons, high intensity red light caused the most severe effect both in complete darkness and ambient light. At first, this seems to be at odds with the sensitivity of rodent retinal opsins for red wavelengths, which peaks at ultraviolet and green spectra and therefore should show little to no activation under red and stronger activation under blue and orange wavelengths. However, it has been recently shown that the rodent retina is more responsive to red light than previously thought, although possibly through the activation of intrinsically photosensitive retinal ganglion cells.^16^ In addition, rodent opsin spectral properties still show a red-shifted absorption, albeit a 10- to 100-fold less for 637 nm compared to green (∼500 nm^31^^,^^32^). Therefore, the reduced retinal sensitivity for red could be compensated by less scattering of red light through brain tissue compared to orange/blue light, which yields a sufficient illumination intensity when reaching the retina. Indeed, red-shifted wavelengths scatter less through media including brain tissue, thereby traveling further and preserving higher irradiance compared to orange and blue light.^15^^,^^17^^,^^18^^,^^19^^,^^20^ In line with this, periorbital ERG recordings have shown that illumination of the retina from an optical fiber implanted in the brain is 50-fold greater when red wavelengths are used compared to shorter wavelengths. Consequently, both the retinal responses measured in a previous study^15^ as well as the cortical response latencies measured in our study are shorter under red light when compared to orange/blue light stimulation.

In the current study, we observed strong spiking responses within the primary and secondary visual cortex to intracranial light stimulation. Given the intra and interhemispheric connectivity of the visual cortex,^33^ as an example, it is very likely that neuronal activation caused by laser stimulation within the brain impacts many areas distant from primary visual pathways. It is expected that off-target activation via subthreshold activity will have a far reaching impact within downstream circuits making this problem even more widespread. It therefore seems best practice to test optogenetic experimental protocols in the absence of exogenous opsins regardless of the identity of the brain area under investigation.

Finally, we show that brain laser stimulation in darkness consistently evokes unintended neuronal activation, while leveraging retinal light-adaptation by exposing the animal to relatively dim ambient light offers a practical and easily implementable solution. We show that this approach is effective under relatively high laser stimulation intensities (particularly for blue/orange wavelengths), while maintaining a low ambient illumination. Factors such as the duration, intensity, and wavelength, as well as the fiber implantation site and the proximity of both the stimulation and recording site to the eyes and visual pathway, should be carefully considered prior to establishing optogenetic experiments.

### Limitations of the study

In this study, we performed extracellular recordings in the visual cortex of mice and found strong off-target effects when using optical fiber stimulation. These data are limited in the sense we have only recorded spiking activity and therefore the potential subthreshold effects on downstream circuits remain unknown. In addition, whole brain wide field imaging with single cell resolution would perhaps provide a more complete overview regarding the extent of off-target activation.

## Resource availability

### Lead contact

Further information and requests for resources should be directed to Troy W. Margrie (t.margrie@ucl.ac.uk).

### Materials availability

This study did not generate new materials.

### Data and code availability

• Preprocessed datasets are available on Figshare: https://doi.org/10.6084/m9.figshare.27109249.v1.
• Analysis code has been deposited on Zenodo: https://doi.org/10.5281/zenodo.13842774.
• Any additional information and raw data required to reanalyze the data reported in this paper are available from the lead contact upon request.

## Acknowledgments

The authors are grateful to the support staff of the Neurobiological Research Facility at Sainsbury Wellcome Center and Manuel Teichert for comments on the manuscript. This work was funded by grants to T.W.M (Wellcome Trust, 219627/Z/19/Z; 214333/Z/18/Z and 10.13039/501100000324Gatsby Charitable Foundation, GAT3755). S.W. was funded by a Feodor-Lynen fellowship from the 10.13039/100005156Alexander von Humboldt Foundation.

## Author contributions

S.W., M.F.V., and T.W.M conceived the project. S.W. and M.F.V. performed all experiments. S.W. analyzed all data. S.W., M.F.V., and T.W.M interpreted and discussed all data and wrote the manuscript.

## Declaration of interests

The authors declare no competing interest.

## STAR★Methods

### Key resources table

**Table:** 

REAGENT or RESOURCE	SOURCE	IDENTIFIER
**Deposited data**		

Preprocessed data structure	Figshare	RRID:SCR_004328; https://doi.org/10.6084/m9.figshare.27109249.v1
Custom analysis code	GitHub	SCR_002630; https://doi.org/10.5281/zenodo.13842774

**Experimental models: Organisms/strains**		

Mouse C57BL/6	Charles Rivers Laboratories	N/A

**Software and algorithms**		

MATLAB 2024	Mathworks	RRID:SCR_001622; https://www.mathworks.com/products/matlab.html
Python (version 3.7.9)	Python Software Foundation	RRID:SCR_008394; https://www.python.org/

**Other**		

637 nm laser source	Thorlabs	S4FC637
594 nm laser source	Coherent	OBIS 594 nm LS 60 mW
473 nm laser source	Coherent	OBIS 473 nm LX 150 mW

### Experimental model and study participant details

All experiments were performed on 20–36 weeks old C57BL/6 male mice in accordance with the UK Home Office regulations (Animal (Scientific Procedures) Act 1986), approved by the Animal Welfare and Ethical Review Body (AWERB; Sainsbury Wellcome Center for Neural Circuits and Behavior) and in compliance with ARRIVE guidelines. Every effort was made to minimize the number of animals and their suffering.

### Method details

#### Surgical procedures

Mice were anesthetized under isoflurane (2%–5%), carprofen or meloxicam was administered (5 mg/kg or 1–2 mg/kg, respectively; s.c.) and eyes were protected with eye gel Lubrithal (Dechra). Mice were then fixed to a stereotaxic frame and their body temperature maintained at 37°C–38°C. They were implanted with a head plate fixed to the skull using Histoacryl (Braun Medical) and C&B Metabond (Sun Medical). Additionally, we added a layer of black dental cement (kemdent) on top of the Metabond. For the implantation of the optical fiber, a craniotomy of ∼1 mm radius was drilled over the region of interest using a 0.3 mm burr dental drill (Osada Electric). The optical cannula (Newdoon 200 microns, 1.5 mm, NA 0.37) was then inserted 550 μm from the pial surface into the primary visual cortex and fixed to the skull using light-cured resin dental cement Relyx Unicem 2 (3M). The skull was then covered with C&B Metabond and subsequently covered with black cement. Animals were allowed to recover for at least 48 h. On the day of extracellular recording (after habituation to head-fixation that typically took 2–3 sessions of 30 min each), animals were anesthetized under isoflurane (2%–5%), their whiskers trimmed (to 2 to 5 mm long) to avoid any whisker related proprioception and a small craniotomy (1 × 1 mm) was drilled over the brain region of interest using a 0.3 mm dental drill. The craniotomy was sealed with silicon kwik-cast (World Precision Instruments) and the animals were allowed to recover for at least 2 h previous to recording.

#### Experimental setup

The visual stimulus was presented on two portable monitors (300 fps; MG300, Magedok). Screens were placed 6 cm from each eye at a 90° angle to each other (visual field covered: ∼75° of elevation and ∼270° azimuth). In addition, an LED strip (Goove) surrounding the experimental setup was turned on (∼1 m away from the animal). The brightness was measured with a Luxmeter (ILM 1337, Isotech) at the position of the eye of the animal. In ambient light experimental conditions, isoluminant gray screens with different brightness (20, 40 and 80 lux) were presented using the Psychophysics Toolbox.^34^ In complete darkness conditions, the screens and LED strip were turned OFF and two small eye cups were positioned gently on the fur surrounding the eyes. In both light and dark conditions, a light shield was positioned at the base of the optical fiber. A Faraday cage was built around the experimental apparatus to provide both electrical noise isolation and pitch darkness.

#### Extracellular recordings

The Neuropixels 2.0 (4 shanks) was first coated with DiI (Molecular Probes, Thermo Fisher Scientific). The probe was then positioned with an angle of 45° to the brain surface and the tip inserted 1500 μm from the pial surface using micro-manipulators (Luigs and Neumann) in either the primary or secondary visual cortex. The probe was allowed to settle for approximately 30 min before recordings. The reference electrode consisted of a Ag/AgCl wire positioned close (<2 mm) to the craniotomy. The craniotomy, as well as the reference wire, were covered by Agar 3% prepared in cortex buffer (NaCl 125 mM, KCl 5 mM, Glucose 10 mM, HEPES 10 mM, CaCl2 2 mM, MgSO4 2 mM, pH 7.4). The Agar was then submerged by cortex buffer. The Neuropixels probe was connected to a PXIe card inside a National Instruments chassis. SpikeGLX v20201103-phase30 (https://github.com/billkarsh/SpikeGLX) was used to acquire data. Data was filtered at 300 Hz during or after acquisition.

#### Laser stimulation

An optical cord (Newdoon MM200/220 NA 0.37) was used to provide the laser stimulation (637 nm: Thorlabs S4FC637; 594 nm: Coherent OBIS 594 nm LS 60 mW; 473 nm: Coherent OBIS 473 nm LX 150 mW). In pitch darkness and ambient light with gray screens, 10 laser stimulations were presented in 5 blocks of increasing laser intensities (1, 2.5, 5, 10 and 15 mW). Within each block, each laser stimulation lasted 0.5 s and was separated by 4 s. Each block was separated by 14 s. Laser power was measured after the fiber tip using a digital power meter (PM100D, Thorlabs). Irradiance was calculated using: http://web.stanford.edu/group/dlab/cgi-bin/graph/chart.php.

#### Probe localization

Following silicon probe recordings, animals were deeply anesthetized and transcardially perfused with cold phosphate buffer (PB, 0.1 M) followed by 4% paraformaldehyde (PFA) in PB (0.1 M) and brains left overnight in 4% PFA at 4°C. Brains were then embedded in 4% agar and imaged using serial two-photon tomography,^35^ using a custom system controlled by ScanImage (Vidrio Technologies) and BakingTray.^36^ Images were acquired as tiles with 5–10 μm axial sampling and 2.27 x 2.27–4.13 × 4.13 μm pixels, and stitched using StitchIt.^37^ Images were then registered to the Allen Mouse Brain Common Coordinate Framework version 3 (CCFv3,^38^ 25 μm resolution) using brainreg.^39^^,^^40^⁠ The atlas data was provided by the BrainGlobe Atlas API.^41^ Probe tracks were confirmed by DiI fluorescence and were traced using brainreg-segment.^39^

#### Spike sorting and analysis

Spike sorting and unit quality control were performed using a fork of the ecephys_spike_sorting pipeline^42^ modified for SpikeGLX data (https://github.com/jenniferColonell/ecephys_spike_sorting; for Neuropixels 2.0 commit 60c40251ed568fb036b4364e615a261d3afc4800). Raw data acquired with SpikeGLX were filtered with CatGT (https://billkarsh.github.io/SpikeGLX/#catgt) and Kilosort2 was run. For single unit analysis only clusters deemed “good” were considered (https://github.com/MouseLand/Kilosort; commit 2a399268d6e1710f482aed5924ba90d52718452a). Double counted spikes were removed.^42^ All units (wide- and narrow-spiking units) were pulled for further analysis.

Firing rates during baseline and laser stimulation periods were calculated on a trial-by-trial basis. Spike trains were convolved with a 5 ms-wide Gaussian window, to obtain a continuous spike rate.

Laser-responsive units were identified using the ZETA (Zenith of Event-based Time-locked Anomalies) test.^43^ The ZETA test is a parameter-free statistical test that enables testing whether neurons show a time-dependent modulation of their firing rates by an event. The ZETA test was restricted to the period during the 0.5 s laser stimulation and ran with 100 random re-samplings to identify which neurons showed significantly light-modulated spiking activity (*p* < 0.05). To estimate a false positive percentage of the ZETA test, we applied the ZETA test on a control period without any laser stimulation. The ZETA test was restricted to a 0.5 s period and performed ten times before and between each stimulation block. Since units recorded in the primary and secondary visual cortices responded similarly to off-target stimulation these units were pooled.

To calculate the change in firing rate two windows of interest of 200 ms and 500 ms were taken: immediately before (baseline, 200 ms) and immediately after laser stimulation onset (response, 500 ms). The average of the spike rate trace during baseline and response window were then subtracted.

### Quantification and statistical analysis

#### Statistics

Details of all n and statistical analysis are provided either in the results and/or in the figure legends. Before comparison of data, individual datasets were checked for normality using the Anderson-Darling test in MATLAB 2024. Statistical analyses were performed using MATLAB 2024. Asterisks indicate significance values as follows: ∗*p* < 0.05, ∗∗*p* < 0.01, ∗∗∗*p* < 0.001.

## References

[1] Nagel G., Szellas T., Huhn W., Kateriya S., Adeishvili N., Berthold P., Ollig D., Hegemann P., Bamberg E. (2003). Channelrhodopsin-2, a directly light-gated cation-selective membrane channel. Proc. Natl. Acad. Sci. USA.

[2] Boyden E.S., Zhang F., Bamberg E., Nagel G., Deisseroth K. (2005). Millisecond-timescale, genetically targeted optical control of neural activity. Nat. Neurosci..

[3] Scanziani M., Häusser M. (2009). Electrophysiology in the age of light. Nature.

[4] Emiliani V., Entcheva E., Hedrich R., Hegemann P., Konrad K.R., Lüscher C., Mahn M., Pan Z.-H., Sims R.R., Vierock J., Yizhar O. (2022). Optogenetics for light control of biological systems. Nat. Rev. Methods Primers.

[5] Packer A.M., Roska B., Häusser M. (2013). Targeting neurons and photons for optogenetics. Nat. Neurosci..

[6] Fenno L., Yizhar O., Deisseroth K. (2011). The development and application of optogenetics. Annu. Rev. Neurosci..

[7] Tsai H.-C., Zhang F., Adamantidis A., Stuber G.D., Bonci A., de Lecea L., Deisseroth K. (2009). Phasic firing in dopaminergic neurons is sufficient for behavioral conditioning. Science.

[8] Huber D., Petreanu L., Ghitani N., Ranade S., Hromádka T., Mainen Z., Svoboda K. (2008). Sparse optical microstimulation in barrel cortex drives learned behaviour in freely moving mice. Nature.

[9] Klapoetke N.C., Murata Y., Kim S.S., Pulver S.R., Birdsey-Benson A., Cho Y.K., Morimoto T.K., Chuong A.S., Carpenter E.J., Tian Z. (2014). Independent optical excitation of distinct neural populations. Nat. Methods.

[10] Bauer J., Weiler S., Fernholz M.H.P., Laubender D., Scheuss V., Hübener M., Bonhoeffer T., Rose T. (2021). Limited functional convergence of eye-specific inputs in the retinogeniculate pathway of the mouse. Neuron.

[11] Hooks B.M. (2018). Dual channel photostimulation for independent excitation of two populations. Curr. Protoc. Neurosci..

[12] Vierock J., Rodriguez-Rozada S., Dieter A., Pieper F., Sims R., Tenedini F., Bergs A.C.F., Bendifallah I., Zhou F., Zeitzschel N. (2021). BiPOLES is an optogenetic tool developed for bidirectional dual-color control of neurons. Nat. Commun..

[13] Adelsberger H., Grienberger C., Stroh A., Konnerth A. (2014). In vivo calcium recordings and channelrhodopsin-2 activation through an optical fiber. Cold Spring Harb. Protoc..

[14] Tsakas A., Tselios C., Ampeliotis D., Politi C.T., Alexandropoulos D. (2021). (INVITED)review of optical fiber technologies for optogenetics. Results Opt.

[15] Danskin B., Denman D., Valley M., Ollerenshaw D., Williams D., Groblewski P., Reid C., Olsen S., Blanche T., Waters J. (2015). Optogenetics in mice performing a visual discrimination task: measurement and suppression of retinal activation and the resulting behavioral artifact. PLoS One.

[16] Niklaus S., Albertini S., Schnitzer T.K., Denk N. (2020). Challenging a myth and misconception: red-light vision in rats. Animals..

[17] Yaroslavsky A.N., Schulze P.C., Yaroslavsky I.V., Schober R., Ulrich F., Schwarzmaier H.J. (2002). Optical properties of selected native and coagulated human brain tissues in vitro in the visible and near infrared spectral range. Phys. Med. Biol..

[18] Yizhar O., Fenno L.E., Davidson T.J., Mogri M., Deisseroth K. (2011). Optogenetics in neural systems. Neuron.

[19] Jacques S.L. (2013). Optical properties of biological tissues: a review. Phys. Med. Biol..

[20] Lehtinen K., Nokia M.S., Takala H. (2021). Red light optogenetics in neuroscience. Front. Cell. Neurosci..

[21] Aravanis A.M., Wang L.-P., Zhang F., Meltzer L.A., Mogri M.Z., Schneider M.B., Deisseroth K. (2007). An optical neural interface: in vivo control of rodent motor cortex with integrated fiberoptic and optogenetic technology. J. Neural. Eng..

[22] Madisen L., Mao T., Koch H., Zhuo J.m., Berenyi A., Fujisawa S., Hsu Y.-W.A., Garcia A.J., Gu X., Zanella S. (2012). A toolbox of Cre-dependent optogenetic transgenic mice for light-induced activation and silencing. Nat. Neurosci..

[23] Olsen S.R., Bortone D.S., Adesnik H., Scanziani M. (2012). Gain control by layer six in cortical circuits of vision. Nature.

[24] Pinto L., Goard M.J., Estandian D., Xu M., Kwan A.C., Lee S.-H., Harrison T.C., Feng G., Dan Y. (2013). Fast modulation of visual perception by basal forebrain cholinergic neurons. Nat. Neurosci..

[25] Chuong A.S., Miri M.L., Busskamp V., Matthews G.A.C., Acker L.C., Sørensen A.T., Young A., Klapoetke N.C., Henninger M.A., Kodandaramaiah S.B. (2014). Noninvasive optical inhibition with a red-shifted microbial rhodopsin. Nat. Neurosci..

[26] Araragi N., Alenina N., Bader M. (2022). Carbon-mixed dental cement for fixing fiber optic ferrules prevents visually triggered locomotive enhancement in mice upon optogenetic stimulation. Heliyon.

[27] Steinmetz N.A., Aydin C., Lebedeva A., Okun M., Pachitariu M., Bauza M., Beau M., Bhagat J., Böhm C., Broux M. (2021). Neuropixels 2.0: A miniaturized high-density probe for stable, long-term brain recordings. Science.

[28] Allen B.D., Singer A.C., Boyden E.S. (2015). Principles of designing interpretable optogenetic behavior experiments. Learn. Mem..

[29] Christie I.N., Wells J.A., Southern P., Marina N., Kasparov S., Gourine A.V., Lythgoe M.F. (2013). fMRI response to blue light delivery in the naïve brain: implications for combined optogenetic fMRI studies. Neuroimage.

[30] Owen S.F., Liu M.H., Kreitzer A.C. (2019). Thermal constraints on in vivo optogenetic manipulations. Nat. Neurosci..

[31] Bridges C.D. (1959). Visual pigments of some common laboratory mammals. Nature.

[32] Peirson S.N., Brown L.A., Pothecary C.A., Benson L.A., Fisk A.S. (2018). Light and the laboratory mouse. J. Neurosci. Methods.

[33] Weiler S., Teichert M., Margrie T.W. (2024). Layer 6 corticocortical cells dominate the anatomical organization of intra and interhemispheric feedback. bioRxiv.

[34] Brainard D.H. (1997). The psychophysics toolbox. Spatial Vis..

[35] Ragan T., Kadiri L.R., Venkataraju K.U., Bahlmann K., Sutin J., Taranda J., Arganda-Carreras I., Kim Y., Seung H.S., Osten P. (2012). Serial two-photon tomography for automated ex vivo mouse brain imaging. Nat. Methods.

[36] Campbell R. (2020).

[37] Campbell R., Blot A., lguerard (2020).

[38] Wang Q., Ding S.-L., Li Y., Royall J., Feng D., Lesnar P., Graddis N., Naeemi M., Facer B., Ho A. (2020). The allen mouse brain common coordinate framework: a 3D reference atlas. Cell.

[39] Tyson A.L., Vélez-Fort M., Rousseau C.V., Cossell L., Tsitoura C., Lenzi S.C., Obenhaus H.A., Claudi F., Branco T., Margrie T.W. (2022). Accurate determination of marker location within whole-brain microscopy images. Sci. Rep..

[40] Niedworok C.J., Brown A.P.Y., Jorge Cardoso M., Osten P., Ourselin S., Modat M., Margrie T.W. (2016). aMAP is a validated pipeline for registration and segmentation of high-resolution mouse brain data. Nat. Commun..

[41] Claudi F., Petrucco L., Tyson A., Branco T., Margrie T., Portugues R. (2020). BrainGlobe Atlas API: a common interface for neuroanatomical atlases. J. Open Source Softw..

[42] Siegle J.H., Jia X., Durand S., Gale S., Bennett C., Graddis N., Heller G., Ramirez T.K., Choi H., Luviano J.A. (2021). Survey of spiking in the mouse visual system reveals functional hierarchy. Nature.

[43] Montijn J.S., Seignette K., Howlett M.H., Cazemier J.L., Kamermans M., Levelt C.N., Heimel J.A. (2021). A parameter-free statistical test for neuronal responsiveness. Elife.

