# Supplementary material for: Overcoming off-target optical stimulation-evoked cortical activity in the mouse brain in vivo
Source: iScience. 2024 Oct 15;27(11):111152. doi: 10.1016/j.isci.2024.111152 (PMC11543908; doi:10.1016/j.isci.2024.111152)
Supplement: Document S1. Figures S1 and S2 [file mmc1.pdf]

iScience, Volume *27*

Supplemental information

Overcoming off-target optical stimulation-evoked cortical activity in the mouse brain *in vivo*

Simon Weiler, Mateo Velez-Fort, and Troy W. Margrie

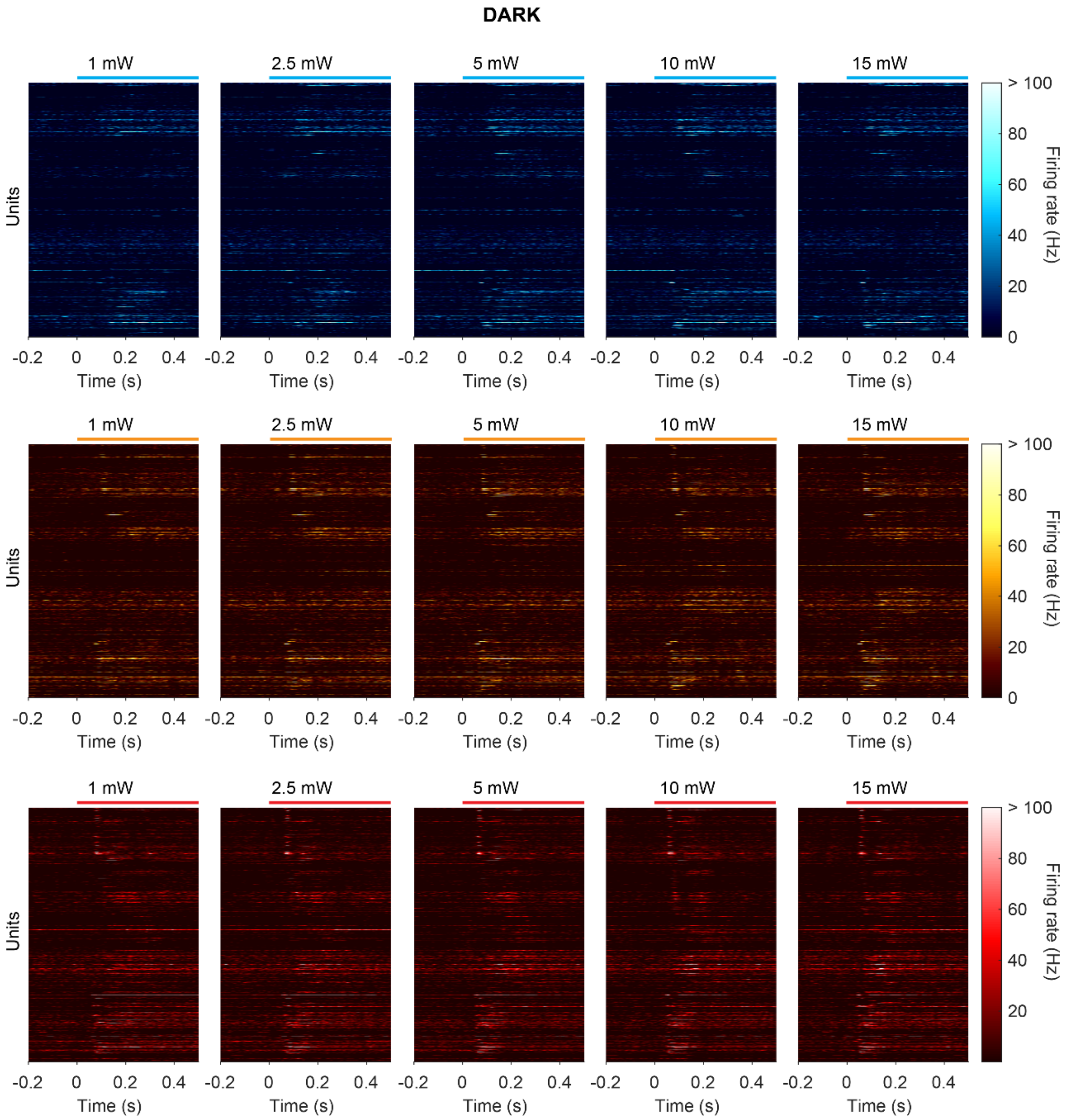

Figure S1: Laser stimulation in the absence of exogenous opsins during extracellular recording in the contralateral visual cortex in darkness, related to Figure 1.

Spike density functions for all units aligned to the onset (stimulation window displayed on top) of blue (top), orange (middle) or red laser stimulation (n=761 cells, n=4 mice) for five laser stimulation intensities in complete darkness

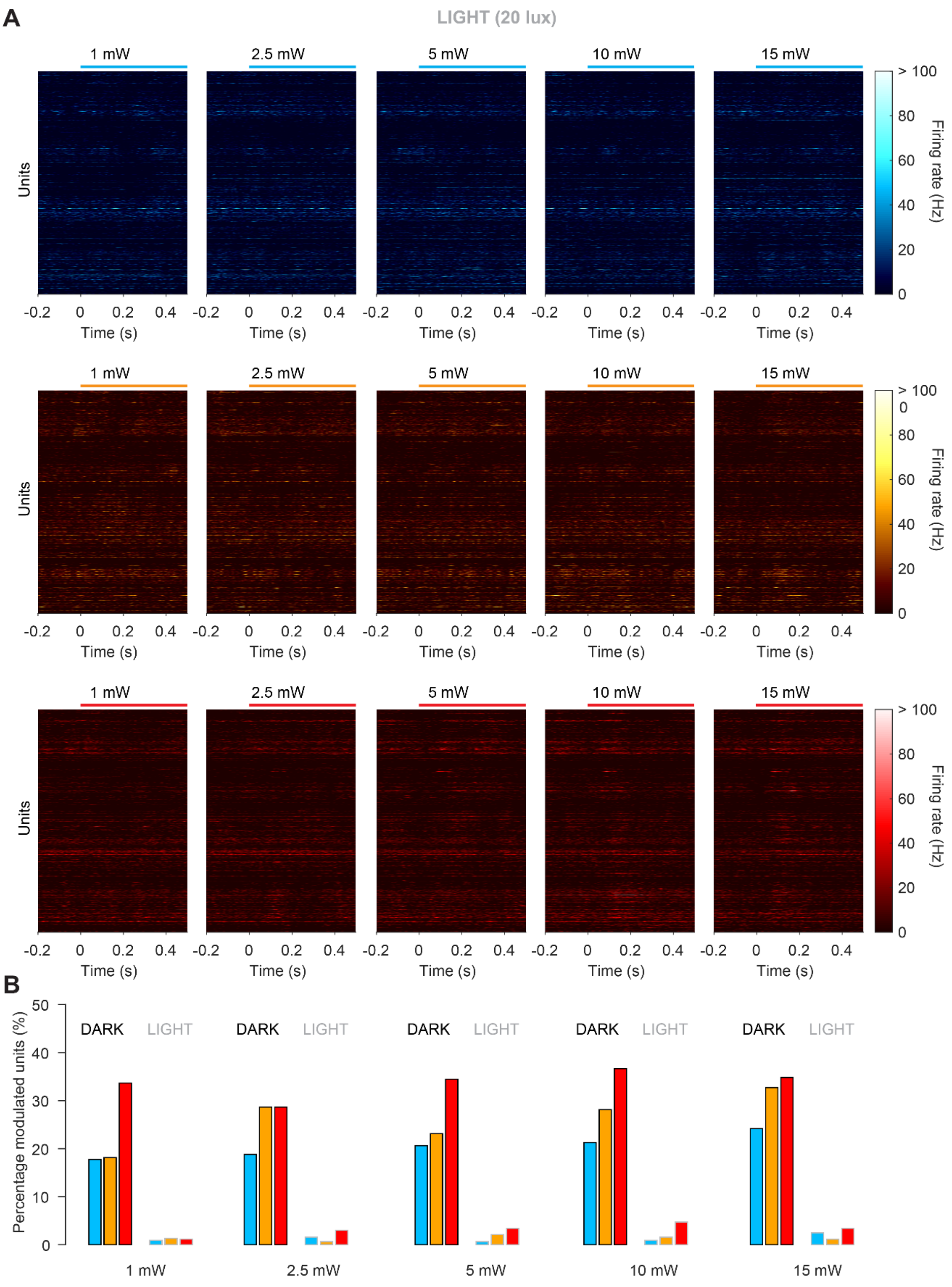

Figure S2: Laser stimulation in the absence of exogenous opsins during extracellular recording in the contralateral visual cortex under ambient light conditions, related to Figure 2.

(A) Spike density functions for all units aligned to the onset (stimulation window displayed on top) of blue (top), orange (middle) or red laser stimulation (n=761 cells, n=4 mice) for five laser stimulation intensities under ambient light of 20 lux.

(B) Percentage of modulated units for blue, orange and red laser stimulations at different laser powers in darkness or under 20 lux of ambient light.
